# Integrated bioinformatic analysis and cell line experiments reveal the significant role of the novel immune checkpoint TIGIT in kidney renal clear cell carcinoma

**DOI:** 10.3389/fonc.2023.1096341

**Published:** 2023-03-24

**Authors:** Qi-Dong Xia, Bo Li, Jian-Xuan Sun, Chen-Qian Liu, Jin-Zhou Xu, Ye An, Meng-Yao Xu, Si-Han Zhang, Xing-Yu Zhong, Na Zeng, Si-Yang Ma, Hao-Dong He, Yu-Cong Zhang, Wei Guan, Heng Li, Shao-Gang Wang

**Affiliations:** Department and Institute of Urology, Tongji Hospital, Tongji Medical College, Huazhong University of Science and Technology, Wuhan, China

**Keywords:** KIRC, TIGIT, targeted therapy, immunotherapy, molecular docking

## Abstract

**Background:**

T cell immunoglobulin and ITIM domain (TIGIT) is a widely concerned immune checkpoint, which plays an essential role in immunosuppression and immune evasion. However, the role of TIGIT in normal organ tissues and renal clear cell carcinoma is unclear. We aim to identify the critical role of TIGIT in renal clear cell carcinoma and find potential targeted TIGIT drugs.

**Materials and methods:**

Data retrieved from the GTEX database and TCGA database was used to investigate the expression of TIGIT in normal whole-body tissues and abnormal pan-cancer, then the transcriptome atlas of patients with kidney renal clear cell carcinoma (KIRC) in the TCGA database were applied to distinguish the TIGIT related features, including differential expression status, prognostic value, immune infiltration, co-expression, and drug response of sunitinib an anti-PD1/CTLA4 immunotherapy in KIRC. Furthermore, we constructed a gene-drug network to discover a potential drug targeting TIGIT and verified it by performing molecular docking. Finally, we conducted real-time polymerase chain reaction (PCR) and assays for Transwell migration and CCK-8 to explore the potential roles of TIGIT.

**Results:**

TIGIT showed a moderate expression in normal kidney tissues and was confirmed as an essential prognostic factor that was significantly higher expressed in KIRC tissues, and high expression of TIGIT is associated with poor OS, PFS, and DSS in KIRC. Also, the expression of TIGIT was closely associated with the pathological characteristics of the tumor, high expression of TIGIT in KIRC was observed with several critical functions or pathways such as apoptosis, BCR signaling, TCR signaling et al. Moreover, the expression of TIGIT showed a strong positive correlation with infiltration of CD8+ T cells and Tregs and a positive correlation with the drug sensitivity of sunitinib simultaneously. Further Tide ips score analysis and submap analysis reveal that patients with high TIGIT expression significantly show a better response to anti-PD1 immunotherapy. Following this, we discovered Selumetinib and PD0325901 as potential drugs targeting TIGIT and verified the interaction between these two drugs and TIGIT protein by molecular docking. Finally, we verified the essential role of TIGIT in the proliferation and migration functions by using KIRC cell lines.

**Conclusions:**

TIGIT plays an essential role in tumorigenesis and progression in KIRC. High expression of TIGIT results in poor survival of KIRC and high drug sensitivity to sunitinib. Besides, Selumetinib and PD0325901 may be potential drugs targeting TIGIT, and combined therapy of anti-TIGIT and other treatments show great potential in treating KIRC.

## Introduction

T cell immunoglobulin and ITIM domain (TIGIT), first introduced by Yu et al. (1), is a member of the poliovirus receptor (PVR)/nectin family and a subset of the immunoglobulin superfamily. The protein encoded by TIGIT contained an extracellular immunoglobulin variable-set (IgV) domain, a type I transmembrane domain, an intracellular immune receptor tyrosine inhibitory motif (ITIM), and an Immunoglobulin tyrosine tail (ITT) motif (1, 2). Interestingly, once introduced, TIGIT was discovered to inhibit T cell activity (1, 3, 4). Moreover, the expression level of TIGIT on the surface of tumor-infiltrating T cells was discovered to increase fourfold than that on peripheral blood mononuclear cells (PBMC), and further studies reveal that only the expression of TIGIT in CD8^+^ T cell exhaustion increased significantly, and changed synchronously with that of PD-1 (5), indicating that TIGIT and PD1/PD-Ll pathway had a synergistic inhibitory effect on tumor-infiltrating T cells. Furthermore, compared with CD8^+^ T cells that less expressed TIGIT, CD8^+^ T cells expressing TIGIT showed a significantly low expression of TNF α, IFN γ, and IL-2. However, the expression of Annexin V and CD95, which represent apoptosis markers, was significantly increased simultaneously (6). Also, when knocked down the expression of TIGIT in CD8^+^ T cells by siRNA, the expression of Annexin V and CD95 decreased significantly, and the level of TNF α, IFN γ, and IL-2 increased significantly (6). Thus, the expression of TIGIT was considered closely related to the apoptosis of CD8^+^ T cells, and once blocking TIGIT signaling pathway, the apoptosis of CD8^+^ T cells can be reversed to some extent. More importantly, it not only plays a significant inhibitory role in CD8^+^ T cells, but TIGIT was also found combating anti-tumor immunity by influencing nature kill cells (7, 8), antigen-presenting dendritic cells (1, 9), and T regulatory cells (Tregs) (10, 11). Thus, TIGIT has been considered one of the most critical immune checkpoints that more and more researchers and scientists devoted to investigating and developing a novel drug for TIGIT, such as TIGIT monoclonal antibody tiragolumab (12). However, our standing of the TIGIT expression in normal organs and tissues is still unclear because we only focused on the immune cell’s expression in TIGIT.

Kidney cancer is the 6th most common cancer in both sexes and the most common urogenital tumor, accounting for approximately 2-3% of all malignancies and 90% of all diagnosed renal parenchymal malignancies1 (13, 14), claiming 14,830 lives with 73,750 new confirmed cases in the USA in 2020 (13). Kidney renal clear cell carcinoma (KIRC) is the predominant pathological subtype of all kidney cancer, accounting for approximately 85% of renal cancer (15, 16), also considered to be one of the most invasive diseases, which is associated with a high mortality rate in the form of metastasis (17). Although surgical intervention is still the main treatment considering that it is not sensitive to radiation, hormone, and cytotoxic therapy. Besides, tyrosine kinase inhibitors (TKIs) such as sunitinib targeting vascular endothelial growth factor (VEGF) pathway also play an essential role in the current clinical treatment as the first-line targeted therapy (18, 19). Moreover, immunotherapy consisting of anti-PD1/PDL1 or anti-CTLA4 therapy have also shown great performance in the therapy of KIRC (20), especially in combination with VEGF-directed therapy (21). Interestingly, immunotherapy combined therapy has replaced TKI’s first-line targeted therapy as a first-line treatment in the latest 2020 European Association of Urology (EAU) guidelines for clear cell metastatic renal cell carcinoma (cc-mRCC) (22).

KIRC has long been categorized as an immunotherapy-responsive cancer type that belongs to ‘hot tumor’ (18). However, the efficacy of Nivolumab monotherapy in advanced renal cell carcinoma was reported as 16% to 29% (23, 24), and the effective rate of Atezolizumab monotherapy was 15% (25–27). It seems only a small part of patients can benefit from immunotherapy, suggesting that other mechanisms must limit anti-tumor immunity. Whether the TIGIT signaling pathway is the significant immunosuppression and immune evasion mechanism in KIRC is unclear. Thus, we wonder what role TIGIT plays in KIRC and whether it could be a potential therapeutic target in the future. In this study, we first systematically explored the expression of TIGIT in various normal organs of the body, especially in the kidney, and then investigated the differential expression of TIGIT between normal tissues and KIRC tissues, explored the prognostic value and clinical correlation of TIGIT in KIRC, further focused on the TIGIT related functions and pathways, investigate the correlation between TIGIT and tumor-infiltrating immune cells, as well as drug sensitivity, and considered TIGIT as a novel therapeutic target and discovered two potential drugs targeting TIGIT by applying molecular docking technology, which referred to the process that a small molecular is spatially docked into a macromolecular and can evaluate the complementary energy at the binding sites, used for structure-based drug design (28) and finally performed a series of *in vitro* experiments to validate our results.

## Materials and methods

### Data acquisition and sources

The transcriptional expression data of normal tissues from the whole-body’s organs and systems, including both male and female, were retrieved from the GTEX database (29). The expression status of TIGIT between the tumor and normal tissues of whole-body was acquired from the GEPIA database (30). The transcriptional data and corresponding survival information of pan-cancer were downloaded from the UCSC Xena (http://xena.ucsc.edu/). The transcriptome profiles of kidney clear cell carcinoma patients and their corresponding clinical characteristics were downloaded from the TCGA database (https://portal.gdc.cancer.gov/) (31). The different expression status of TIGIT in pan-cancer and the corresponding immune infiltration of each sample emphasized by multiple acknowledged methods was acquired from TIMER 2.0 database (http://timer.cistrome.org/) (32).

### TIGIT in normal tissues between organs and genders or between tumor and normal tissues

The expression of TIGIT in normal tissues from the whole-body was extracted and sorted according to the expression value. Then we visualized it as a boxplot to show the ranking of TIGIT’s expression. Besides, we compared the expression of TIGIT in the same organ tissues but between different genders by performing Wilcoxon rank-sum test. Following this, we visualized the expression of TIGIT in whole-body including male and female by applying R program package ‘gganatogram’. We would also like to investigate the expression status of TIGIT between tumor and normal tissues in the whole-body, especially in the kidney. Thus, we searched TIGIT in pan-cancer from the GEPIA database and acquired the differential expression plot.

### TIGIT in KIRC: Differential expression, prognostic value, and clinical correlation

The fragments per kilobase of per million formats (FPKM) of kidney clear cell carcinoma (KIRC) transcriptome profiles were sorted and normalized. The expression of TIGIT in the KIRC tumor and normal adjacent tumor tissues was extracted. Wilcoxon rank-sum test was performed to compare the differential expression of TIGIT between tumor and normal tissues in KIRC (including both paired and non-paired samples). Following this, samples were divided into high or low TIGIT expression groups by the expression of TIGIT that was higher/lower than the medium value was considered high/low TIGIT expression groups. Then Kaplan-Meier methods survival curves were plotted that including overall survival (OS), progression-free survival (PFS), disease-specific survival (DSS), and disease-free survival (DFS). The log-rank test was also carried out to examine these survival interval differences between high and low TIGIT expression patients. Further univariate and multivariate cox regression was applied to check whether TIGIT could serve as an independent prognostic factor and the differential expression status of TIGIT between different clinicopathological subgroups containing age (<=65 or >65). gender (male or female), grade (G1, G2, G3, G4), grade (G1-2 or G3-4), stage (stage I, stage II, stage III, stage IV), stage (stage I-II or stage III-IV), pathological T stage (T1, T2, T3, T4), pathological N stage (N0 or N1), and pathological M stage (M0 or M1) were compared by Wilcoxon rank-sum test.

### TIGIT in KIRC: Differential enhanced pathways, differential immune infiltration, and differential drug response

Same as above, samples were grouped as high or low TIGIT expression, and the transcriptome profiles were merged, proceeded, and exported as ‘gct’ and ‘cls’ format files prepared for the following gene set enrichment analysis (GSEA). The GSEA version 4.0.3 was applied to perform the enrichment analysis, and here we focused on the HALLMARK gene sets and KEGG pathway gene sets. Discovered the enhanced pathways were associated with immunity, and as TIGIT was an immune checkpoint, we were interested in the association between TIGIT and immune infiltration in KIRC. However, there were several acknowledged methods to estimate the immune infiltration of samples according to their transcriptional expression atlas. Thus, here we performed seven different methods to precisely investigate the immune infiltration status of KIRC patients, including XCELL, TIMER, QUANTISEQ, MCPCOUNTER, EPIC, CIBERSORT-ABS, and CIBERSORT. We then applied the SPEARMAN correlation test to explore the significant TIGIT-related immune cells with p < 0.05, we explored the differential immune infiltration between the high-/low-TIGIT group by the Wilcox test. Besides, we were also interested in the drug response of the first-line targeted therapy for renal clear cell carcinoma, applying R program package ‘pRRophetic’ to predict each sample’s drug sensitivity to the targeted therapy of sunitinib. Then compared the different drug sensitivity between high-TIGIT and low-TIGIT patients by using Wilcoxon signed-rank test and explored the correlation between TIGIT and the drug sensitivity by applying the SPEARMAN correlation test to discover the association between expression of TIGIT and drug sensitivity of the targeted therapy. Furthermore, Tide ips scores analysis and submap algorithm were applied to predict the treatment response to anti-PD1 or anti-CTLA4 immunotherapy between KIRC patients with high-/low-TIGIT expression.

### TIGIT in KIRC: Novel potential targeted drug and molecular docking

Interested in the TIGIT and targeted therapy, we searched TIGIT in the IGMDR database (33), acquired the gene-drug network, and discovered two potential targeted therapy drugs for TIGIT. Subsequently, molecular docking was applied to verify the interaction between these two drugs and TIGIT. The 2D structure of these two drugs was acquired from the PubChem database (34), and ChemBio 3D software was used to calculate the 3D structure with minimizing energy. The receptor protein encoded by TIGIT was searched in the Uniprot database (35), and then the 3D structure of the protein was downloaded from the RCSB PDB database (36). PyMOL 2.4.0 software was applied to conduct the dehydration of the receptor protein, and Autodock software was used to carry out further hydrogenation and charge calculation of proteins. Parameters of the receptor protein docking site were set to include the active pocket sites where small-molecule drugs bind. Finally, Autodock Vina was used to conduct docking the receptor protein encoded by TIGIT with the small molecule drugs.

### Cell culture

The human ccRCC cell lines (786-O), the human embryonic kidney 293T (HEK-293T) cell and the human renal tubular epithelial cell lines (HK_2_) were purchased from the Shanghai Cell Bank Type Culture Collection Committee (Shanghai, China). The 786-O and HK_2_ cells were cultured in RPMI-1640 (Gibco, Thermo Fisher Scientific, Waltham, MA, United States) supplemented with 10% FBS and 100 U/mL Penicillin/Streptomycin in a 5% CO2 incubator. While the HEK-293T cells were cultured in high-glucose DMEM media supplemented with 10% FBS. Cells were collected at 90% confluence, and the medium was changed every 48–72 h.

### Cell transfection

Relative target fragments were inserted into lentiviral vectors PCDH-CMV-MCS-EF1-copGFP. Together with pGC-LV, pHelper1.0, pHelper2.0, pHelper3.0, and recombinant lentiviral vectors, plasmids were co-transfected into HEK-293T cells using Lipofectamine 3,000 (Invitrogen, United States).

### RNA extraction and quantitative real-time polymerase chain reaction

Total RNAs of cells or tissues were extracted using the TRIzol reagent (Vazyme, R401-01), and then cDNA was synthesized by reverse transcription using the HiScript III RT SuperMix for qPCR (Vazyme, R323-01). RT-PCR was conducted using Taq Pro Universal SYBR qPCR Master Mix (Vazyme, Q712-02). GAPDH was used as an internal control. **Supplementary Table S1** displayed the sequences of all primers.

### CCK-8 assay

1,500 of 786-O cells were seeded into 96-well plates per well for the CCK-8 assay. Then 10 μL CCK-8 (MCE, HY-K0301) was added to each well for 1-h incubation, and the absorbance of each well was measured at 450 nm every day for 5 times.

### Transwell migration assay

For migration assays, about 5 × 10^4^ of 786-O cells were suspended and seeded in the upper chambers of 24-well transwell plates (Corning, United States) with 250μl FBS-free medium. Then, 500μl RPMI-1640 with 10% FBS was added to the lower chamber. After 12h incubation, the chambers were fixed and stained with crystal violet for 30 min. Finally, imaging was performed under an inverted microscope

## Results

### Basic characteristics

**Figure 1:**
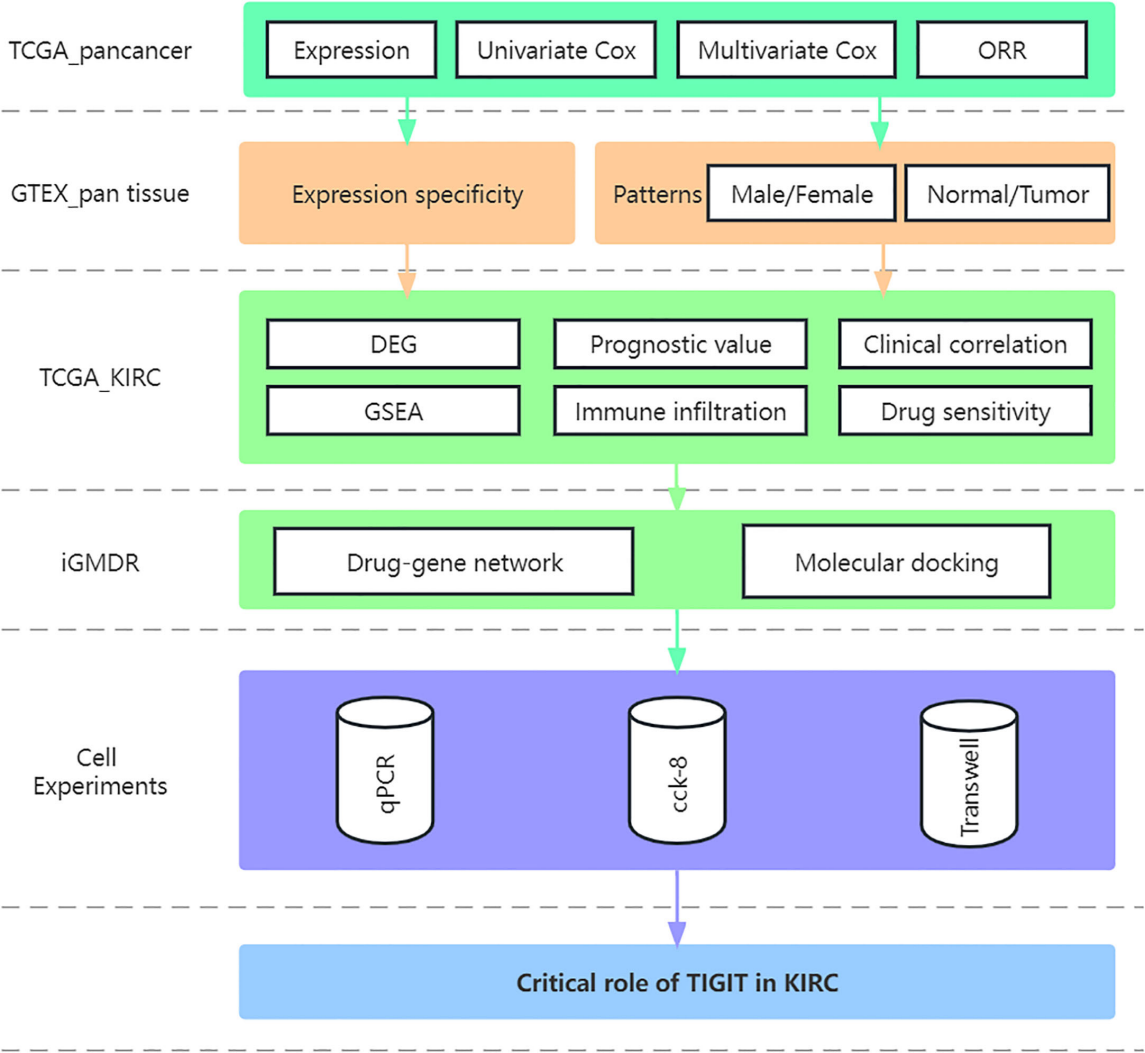
The study flow.

The study flow was displayed in the **Figure 1**. A total of 611 transcriptome profile (72 normal tissue and 539 tumor tissue) from 530 TCGA_KIRC patients were downloaded and sorted, for those samples sequenced multiple time, we took the average of them as their transcriptional data. and the characteristic of the samples were shown in **Table 1**, χ^2^ test or Fisher’s exact test were performed to explore the heterogeneity between high or low expression of TIGIT.

**Table 1:** Detailed clinicopathological characteristics of the KIRC patients.

	Overall	High TIGIT	Low TIGIT	P-value
Number	530	265	265	
Age (mean (SD))	60.56 (12.14)	60.42 (11.81)	60.71 (12.47)	0.789
Gender = FEMALE/MALE (%)	186/344 (35.1/64.9)	81/184 (30.6/69.4)	105/160 (39.6/60.4)	0.036
Grade (%)				<0.001
G1	14 (2.6)	4 (1.5)	10 (3.8)	
G2	227 (42.8)	91 (34.3)	136 (51.3)	
G3	206 (38.9)	117 (44.2)	89 (33.6)	
G4	75 (14.2)	52 (19.6)	23 (8.7)	
GX	5 (0.9)	0 (0.0)	5 (1.9)	
unknow	3 (0.6)	1 (0.4)	2 (0.8)	
Stage (%)				<0.001
Stage I	265 (50.0)	106 (40.0)	159 (60.0)	
Stage II	57 (10.8)	34 (12.8)	23 (8.7)	
Stage III	123 (23.2)	73 (27.5)	50 (18.9)	
Stage IV	82 (15.5)	50 (18.9)	32 (12.1)	
unknow	3 (0.6)	2 (0.8)	1 (0.4)	
T (%)				<0.001
T1	21 (4.0)	6 (2.3)	15 (5.7)	
T1a	140 (26.4)	45 (17.0)	95 (35.8)	
T1b	110 (20.8)	60 (22.6)	50 (18.9)	
T2	55 (10.4)	29 (10.9)	26 (9.8)	
T2a	10 (1.9)	7 (2.6)	3 (1.1)	
	4 (0.8)	4 (1.5)	0 (0.0)	
T3	5 (0.9)	4 (1.5)	1 (0.4)	
T3a	120 (22.6)	68 (25.7)	52 (19.6)	
T3b	52 (9.8)	35 (13.2)	17 (6.4)	
T3c	2 (0.4)	0 (0.0)	2 (0.8)	
T4	11 (2.1)	7 (2.6)	4 (1.5)	
M (%)				0.002
M0	420 (79.2)	206 (77.7)	214 (80.8)	
M1	78 (14.7)	50 (18.9)	28 (10.6)	
MX	30 (5.7)	8 (3.0)	22 (8.3)	
unknow	2 (0.4)	1 (0.4)	1 (0.4)	
N (%)				0.107
N0	239 (45.1)	121 (45.7)	118 (44.5)	
N1	16 (3.0)	12 (4.5)	4 (1.5)	
NX	275 (51.9)	132 (49.8)	143 (54.0)	

### TIGIT in normal tissues and tumor tissues

**Figure 2:**
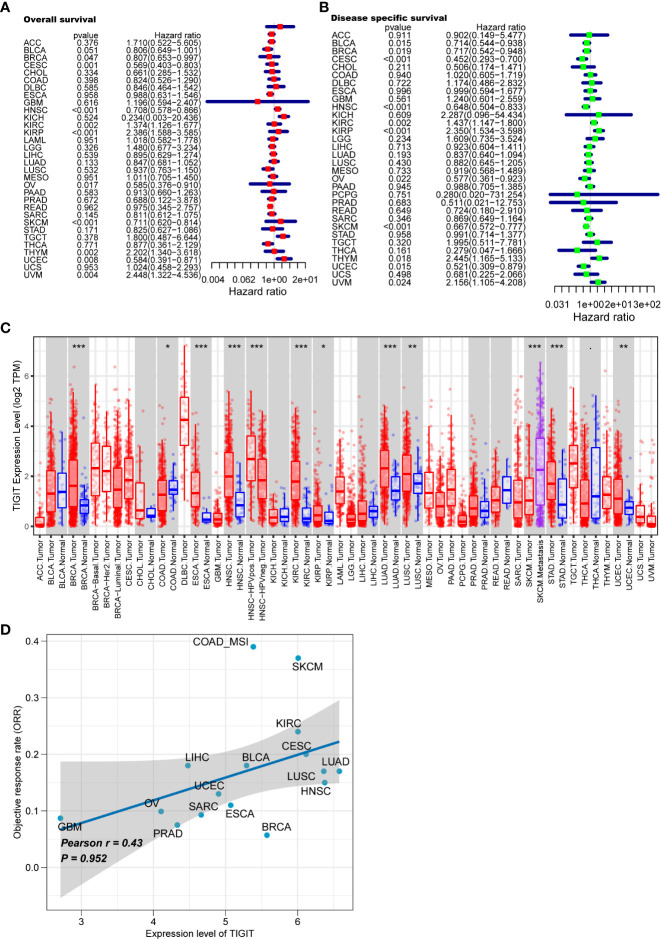
Analysis of TIGIT in pan-cancer. **(A)** Univariate Cox regression showed the OS of TIGIT in pan-cancer. **(B)** Univariate Cox regression showed the disease specific survival of TIGIT in pan-cancer. **(C)** Differential expression status of TIGIT in pan-cancer. **(D)** The potiential association between the expression level of TIGIT and objective response rate in various cancers. *: p<0.05, **: p<0.01, ***: p<0.001

**Figure 3:**
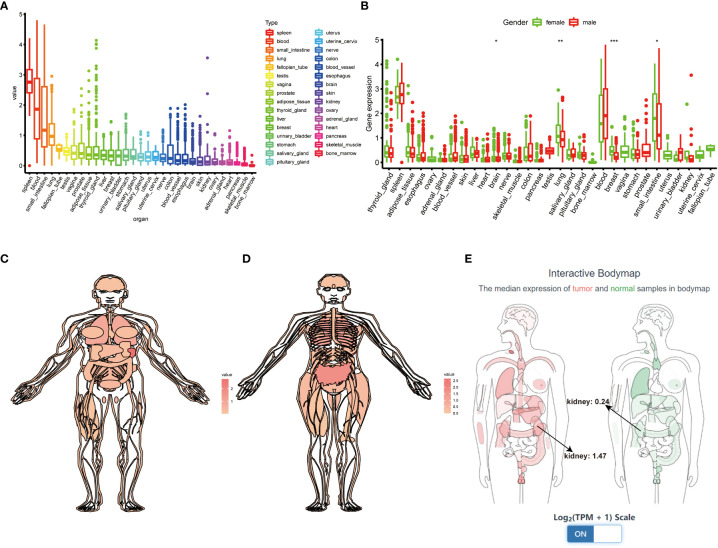
Comprehensive analysis of TIGIT in whole-body’s normal tissues. **(A)** The expression status of TIGIT in normal organs sorted by the expression value. **(B)** The differential expression status of TIGIT between males and females. **(C)** The expression atlas of TIGIT in males. **(D)** The expression atlas of TIGIT in females. **(E)** The expression of TIGIT in tumor organ tissues (red) and normal organ tissues (green). *: p<0.05, **: p<0.01, ***: p<0.001

We first systematically analyze the relationship between TIGIT and a variety of cancers, especially kidney cancer, and discovered that the expression of TIGIT was quite high in KIRC, but not KICH and KIRP, and was associated with poor prognosis (**Figures 2A–C**). We also found that there is a positive correlation between the expression level of TIGIT and objective response rate (ORR) in various cancers (**Figure 2D**). And the first three organs with the highest expression of TIGIT were the spleen, blood, and small intestine. The lowest three were pancreas, skeletal muscle, and bone marrow, and TIGIT showed a moderate expression in normal kidney (**Figure 3A**). Interestingly, the expression of TIGIT in females’ brains, lungs, breasts, and small intestine was significantly higher than that in males (**Figure 3B**). TIGIT was the highest expression in the spleen in males and females (**Figures 3C, D**). Here we focused on the kidney and discovered a higher expression of TIGIT in kidney tumor with a mean expression of 0.24 in normal kidney and that of 1.47 in kidney tumor (**Figure 3E**).

### TIGIT in KIRC: Differential expression, prognostic value, and clinical correlations

**Figure 4:**
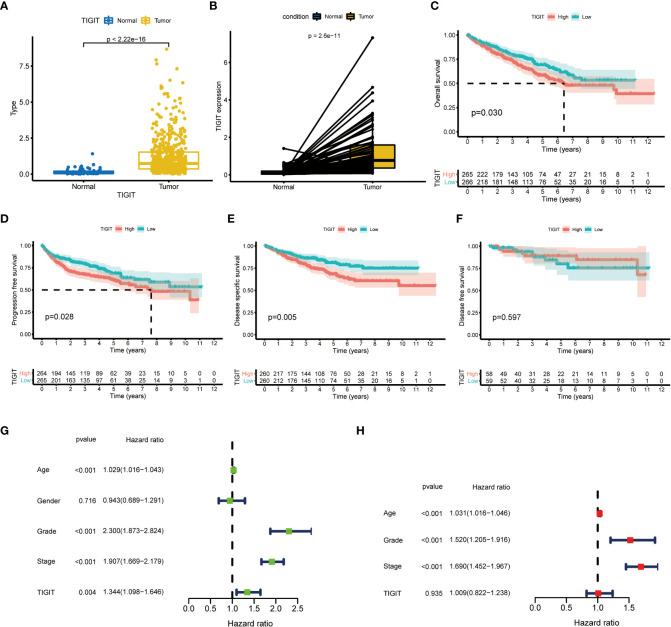
Differential expression and prognostic value of TIGIT in KIRC. **(A)** TIGIT shows a significantly higher expression in non-paired KIRC tissues compared to the normal tissues. **(B)** TIGIT shows a significantly higher expression in paired KIRC tissues compared to the normal tissues. **(C)** High expression of TIGIT was associated with significantly poor overall survival in KIRC. **(D)** High expression of TIGIT was associated with significantly poor progression-free survival in KIRC. **(E)** High expression of TIGIT was associated with significantly poor disease-specific survival in KIRC. **(F)** There were no significant differences between patients with high or low expression of TIGIT in disease-free survival in KIRC. **(G)** Univariate Cox regression showed TIGIT a significant prognostic factor in KIRC. **(H)** Multivariate Cox regression of TIGIT in KIRC.

**Figure 5:**
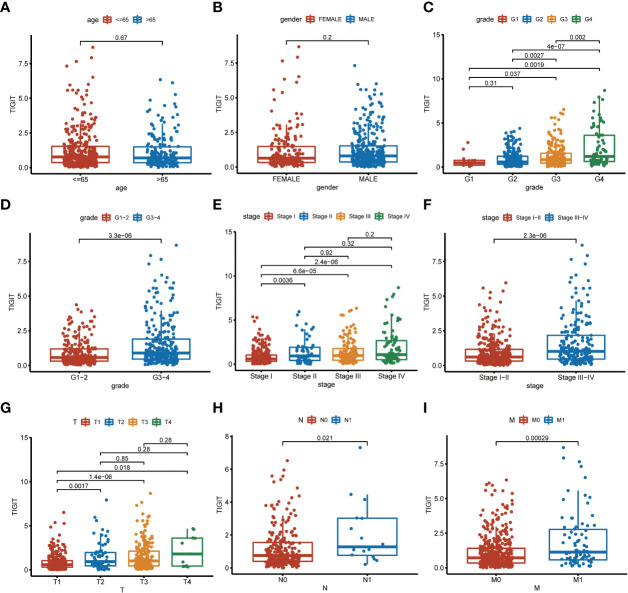
Clinical correlation of TIGIT expression. **(A)** clinical correlation between age and TIGIT. **(B)** clinical correlation between gender and TIGIT. **(C)** clinical correlation between grade and TIGIT. **(D)** clinical correlation between grade and TIGIT. **(E)** clinical correlation between stage and TIGIT. **(F)** clinical correlation between stage and TIGIT. **(G)** clinical correlation between T stage and TIGIT. **(H)** clinical correlation between N stage and TIGIT. **(I)** clinical correlation between M stage and TIGIT.

TIGIT showed a significantly higher expression in KIRC tissues than normal tissues in both non-paired and paired samples (**Figures 4A, B**). Following this, we wondered whether high expression of TIGIT resulted in poor clinical outcomes and discovered the high expression of TIGIT was associated with poor overall survival (**Figure 4C**), poor progression survival (**Figure 3D**), and poor disease-specific survival (**Figure 4E**). There was no difference in disease-free survival (**Figure 4F**). This showed that TIGIT played an essential role in the tumorigenesis, progression, and clinical outcomes of KIRC. Besides, we performed univariate and multivariate cox regression and found TIGIT as a significant risk factor with a hazard ratio (HR) of 1.344 (1.098 to 1.646) for KIRC patients in univariate Cox regression (**Figure 4G**). Subsequently, after correction from other clinical features, the HR of TIGIT was 1.009 (0.822 to 1.238), showing no significant difference (**Figure 4H**). This suggested that the expression of TIGIT was significant associated with clinical characteristics, so we conducted further exploration about the clinical correlation of TIGIT. There were no significant differences between age (**Figure 5A**) and gender (**Figure 5B**). However, TIGIT showed great association with pathological characteristics as expected. TIGIT showed a gradually increasing trend from G1 to G4 (**Figure 5C**), and significantly higher expressed in G3-4 than G1-2 (**Figure 5D**). Also showed the same trend from Stage I to Stage IV (**Figure 5E**), and significantly higher expressed in Stage III-IV than Stage I-II (**Figure 5F**). Besides, TIGIT was significantly lowest expressed in T1 than T2 to T4 (**Figure 5G**), and significantly higher expressed in N1 than N0 (**Figure 5H**), in M1 than M0 (**Figure 5I**), which showed the significant role of TIGIT in the tumor metastasis.

### TIGIT in KIRC: Differential enhanced pathways, differential immune infiltration, and differential drug response

**Figure 6:**
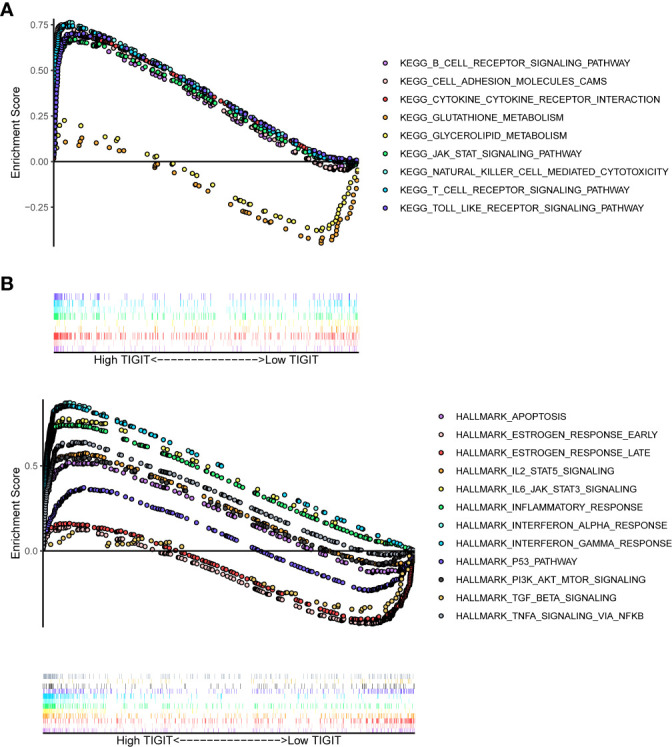
Differential enriched functions or pathways correlated with the expression of TIGIT. **(A)** Differential enriched KEGG pathways associated with the expression of TIGIT in KIRC. **(B)** Differential enriched HALLMARK pathways associated with the expression of TIGIT in KIRC.

Having identified TIGIT as an essential prognostic factor and explored its association between expression and clinical characteristics, we were interested in the functions and pathways influenced by TIGIT. Subsequent KEGG enrichment analysis showed high expression of TIGIT was associated with significantly enhanced pathways such as B cell receptor signaling pathway, cell adhesion molecular cams, cytokine-cytokine receptor interaction, JAK-STAT signaling pathway, nature kill cell-mediated cytotoxicity, T cell receptor signaling pathway, and Toll-like receptor signaling pathway, also associated with significantly attenuated functions such as glutathione metabolism and glycerolipid metabolism (**Figure 6A**). HALLMARK gene set enrichment analysis suggested high expression of TIGIT was associated with significantly enhanced functions and pathways such as apoptosis, IL2-STAT5 signaling pathways, IL6-JAK-STAT3 signaling, inflammatory response, interferon-α response, interferon-Λ response, P53 pathway, PI3K-AKT-mTOR signaling, and TNF-α signaling *via* NF-κB, and significantly attenuated functions such as estrogen response and TGF beta signaling (**Figure 6B**). It was interesting that TIGIT was associated with so many essential pathways and functions in KIRC.

**Figure 7:**
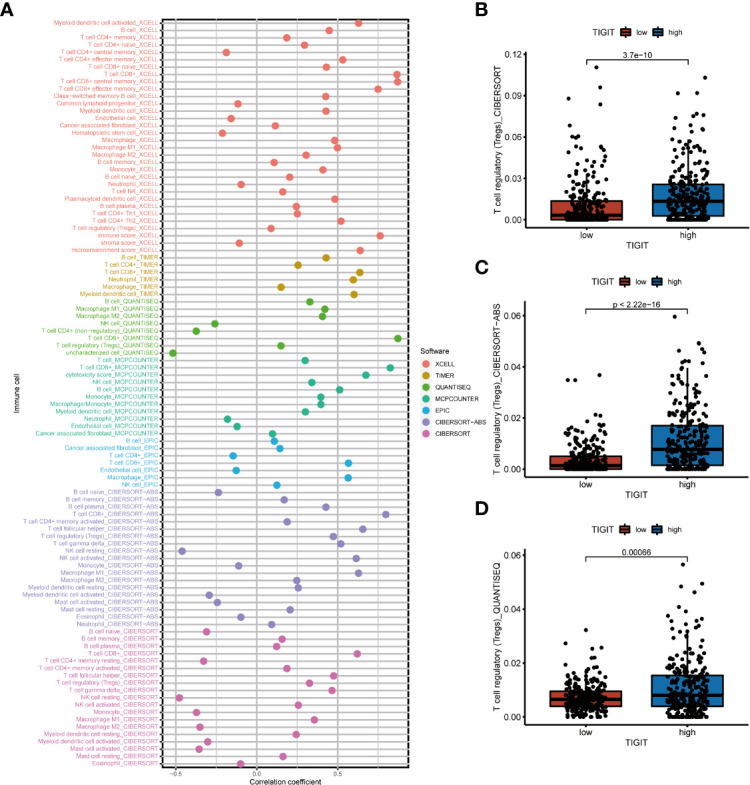
The correlation between immune infiltration and the expression of TIGIT in KIRC. **(A)** Spearman correlation test showed TIGIT was significantly associated with several types of immune infiltration cells. **(B)** Differential infiltration of Tregs between high or low TIGIT expression patients calculated by the CIBESORT. **(C)** Differential infiltration of Tregs between high or low TIGIT expression patients calculated by the CIBESORT-ABS. **(D)** Differential infiltration of Tregs between high or low TIGIT expression patients calculated by the QUANTISEQ.

As TIGIT is one of the most important immune checkpoints associated with so many immunity-related functions and pathways, we further investigated the association between its expression and patients’ immune infiltration. The SPEARMAN correlation test suggested the expression of TIGIT was significant negative correlated with NK resting cell, endothelial cell, neutrophil, M2 macrophages, and significant positive correlated with M1 macrophages, CD8^+^ T cells, T regulatory cells (Tregs), Th1 cells, Th2 cells et al. (**Figure 7A**). All these seven emphasized methods suggested TIGIT a strong positive correlation with CD8^+^ T cells, which should have resulted in a great clinical outcome. So, we focused on the Tregs, and discovered TIGIT was significantly positively correlated with the infiltration of Tregs (**Figure 7A**), and significant-high infiltration with Tregs was observed in high TIGIT expression samples emphasized by CIBESORT (**Figure 7B**), CIBESORT-ABS (**Figure 7C**), and QUANTISEQ (**Figure 7D**).

**Figure 8:**
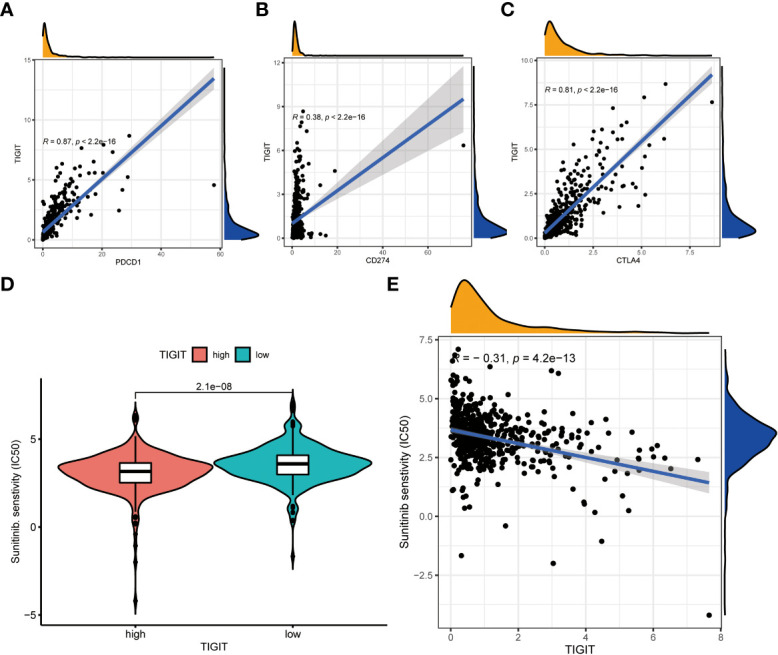
The co-expression between TIGIT and other common immune checkpoints and the drug response of sunitinib between high or low TIGIT expression patients. **(A)** TIGIT was significantly positive co-expression with PDCD1. **(B)** TIGIT was significantly positive co-expression with CD247. **(C)** TIGIT was significantly positive co-expression with CTLA4. **(D)** High expression of TIGIT was associated with a significantly higher drug sensitivity of sunitinib in KIRC. **(E)** TIGIT was significantly positively correlated with the drug sensitivity of sunitinib in KIRC.

**Figure 9:**
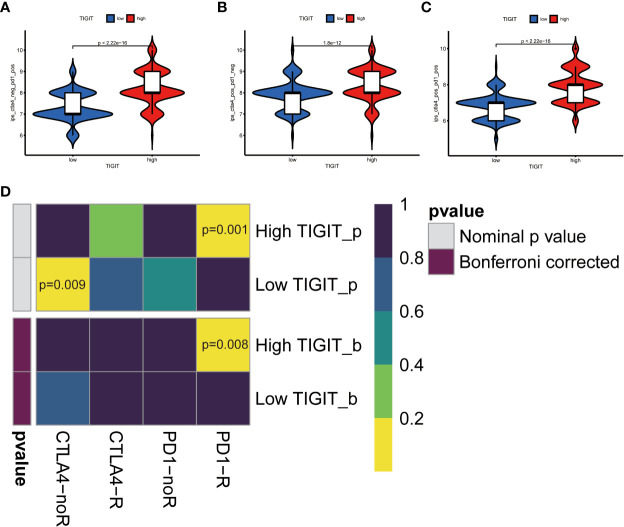
TIGIT and response of immunotherapy. **(A)** Prediction of immunotherapy in CTLA4 negative PD1 positive patients with high/low TIGIT expression. **(B)** Prediction of immunotherapy in CTLA4 positive PD1 negative patients with high/low TIGIT expression. **(C)** Prediction of immunotherapy in CTLA4 positive PD1 positive patients with high/low TIGIT expression. **(D)** Prediction of response to anti-PD1 or anti-CTLA4 immunotherapy by submap in KIRC patients with high/low TIGIT expression.

Observed TIGIT as a significant correlation with immune infiltration in KIRC, we were interested in the correlation between TIGIT and other common immune checkpoints such as PD1(PDCD1), PD-L1 (CD274), and CTLA4. As expected, we found TIGIT significant positive correlated with PDCD1 (R =0.87, p< 0.001), CD274 (R=0.38, p< 0.001), CTLA4 (R=0.81, p< 0.001) as **Figures 8A–C**. This may explain the poor response for the existing immunotherapy in KIRC that although we inhibit some immune checkpoints like PD1, PD-L1, or CTLA4, their associated expression of TIGIT still plays a role in immunosuppression and immune evasion. Besides, we further explored the correlation between the expression of TIGIT and the drug response of sunitinib, the most used targeted therapy drug in KIRC. Discovered high expression of TIGIT was associated with a significantly higher response for sunitinib (**Figure 8D**), and TIGIT showed a significant positive correlation with the drug sensitivity of sunitinib (R= -0.31, p< 0.001) as **Figure 8E**. Further Tide ips scores analysis showed that KIRC patients with high TIGIT expression may response better to anti-PD1 immunotherapy (**Figure 9A**), anti-CTLA4 immunotherapy (**Figure 9B**), and combined immunotherapy (**Figure 9C**). Also, the submap analysis reaches a consistent result that KIRC patients with high TIGIT expression showed a significant better response to anti-PD1 immunotherapy (p=0.001, Bonferroni corrected p=0.008, **Figure 9D**).

### TIGIT in KIRC: Novel potential targeted drug and molecular docking

**Figure 10:**
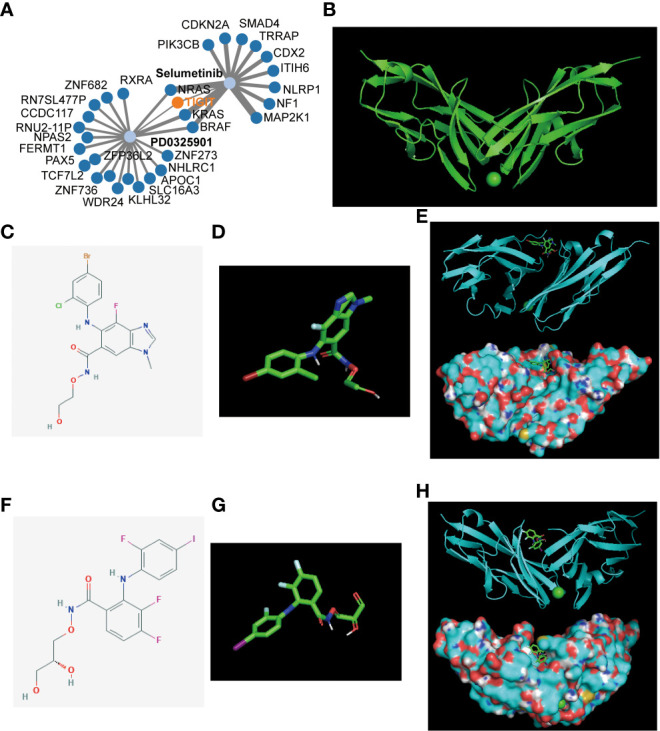
Gene-drug network and molecular docking. **(A)** The potential gene-drug networks target TIGIT. **(B)** The 3D structure of the TIGIT protein. **(C)** The 2D structure of Selumetinib. **(D)** The 3D structure of Selumetinib. **(E)** The molecular docking between Selumetinib and TIGIT showed Selumetinib could enter into the active pocket of TIGIT protein. **(F)** The 2D structure of PD0325901. **(G)** The 3D structure of PD0325901. **(H)** The molecular docking between PD0325901and TIGIT showed PD0325901could enter into the active pocket of TIGIT protein.

After revealing the important role of TIGIT in immunotherapy and targeted therapy of KIRC, we believe that TIGIT is an important therapeutic target for KIRC and intend to discover a new drug or a new use targeting TIGIT in conventional drugs. Thus, we constructed the gene-drug network (**Figure 10A**) and found two potential therapeutic drugs targeting TIGIT, and they were Selumetinib and PD0325901. To verify our discovery, we performed molecular docking technology to examine the interaction between these two drugs and TIGIT protein. The 3D structure of the TIGIT protein was shown in **Figure 10B**, the 2D structure and 3D structure of Selumetinib were shown in **Figures 10C, D**, that of PD0325901 was shown in **Figures 10F, G**. Both molecular dockings for Selumetinib and PD0325901 showed that these two drugs could enter into the active pocket of TIGIT (**Figures 10E, H**), which suggested they could serve as potential drugs targeting TIGIT.

### TIGIT enhanced the progression of 786-O clear cell renal carcinoma cells

**Figure 11:**
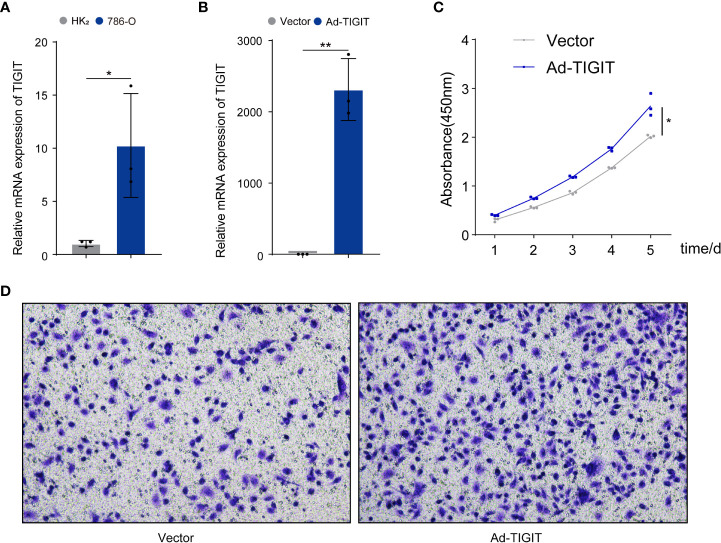
TIGIT affected the proliferation and migration of cells in renal carcinoma. **(A)** qRT-PCR was used to detect expression levels of TIGIT in tumor cells and normal cells. **(B)** The overexpression of TIGIT in 786-O cells was confirmed by qRT-PCR. **(C)** CCK8 assay: TIGIT could increase the viability of 786-O cells. **(D)** Transwell migration assay: TIGIT could promote the migration of 786-O cells. *p < 0.05; **p < 0.01.

Finally, we validated the potential physiological role of TIGIT in *in vitro* experiments. We explored the expression of TIGIT in renal carcinoma cells (786-O) and normal cells (HK_2_) and found that the level of TIGIT in tumor cells was significantly increased compared to normal cells (**Figure 11A**). To investigate the biological functions of TIGIT in renal carcinoma, TIGIT was overexpressed in 786-O cells by lentiviral infection, and its expression was validated by qRT-PCR (**Figure 11B**). CCK8 assay demonstrated that TIGIT promoted cellular viability of 786-O cells by contrast with control groups (**Figure 11C**). Furthermore, we explored whether TIGIT was involved in cell metastasis and discovered that the overexpression of TIGIT remarkably increased migration ability in 786-O cells (**Figure 11D**). Taken together, these findings indicated that TIGIT enhanced carcinogenesis of renal carcinoma cells *in vitro*.

## Discussion

The present study conducted a comprehensive analysis of TIGIT in KIRC, confirmed TIGIT as an essential prognostic factor significantly higher expressed in KIRC tissues, and high expression of TIGIT is associated with a poor OS, PFS, and DSS in KIRC. Also, the expression of TIGIT was closely associated with the pathological characteristics of the tumor, high expression of TIGIT in KIRC was observed with several critical functions or pathways such as apoptosis, BCR signaling, TCR signaling et al. Moreover, the expression of TIGIT shows a strong positive correlation with infiltration of CD8^+^ T cells and Tregs, and shows a positive correlation with the drug sensitivity to sunitinib and anti-PD1 immunotherapy at the same time. Furthermore, we constructed a gene-drug network, discovered Selumetinib and PD0325901 as potential drugs targeting TIGIT, and verified the interaction between these drugs and TIGIT protein by molecular docking. Finally, *in-vitro* experiments verified the essential role of TIGIT in KIRC.

Hong et al. reported a significant positive observation of TIGIT expression in renal cell carcinoma (RCC) tissues than adjacent normal tissues by immunohistochemistry in their cohorts (37), which was consistent with our results TIGIT showed a significantly higher expression in KIRC tissues than normal tissues. Also, Yin et al. reported the prognostic value of TIGIT in KIRC and constructed a survival-predicting model based on this (38). All these studies confirmed the significant role TIGIT played in tumorigenesis, progression, and clinical outcomes of KIRC. Interestingly, not only KIRC, Duan et al. reported TIGIT as an effective tumor biomarker in human hepatocellular carcinoma (HCC) that the expression levels of TIGIT were upregulated in the cancerous tissues with the degree of cancerous differentiation from high to low from patients with HCC, and TIGIT showed positive correlation with the level of α-fetoprotein (AFP), which reveals the potential of TIGIT as a cancer biomarker in HCC (39). Thus, Whether TIGIT is differentially expressed in the pan-cancer spectrum and whether TIGIT can be used as a tumor marker of pan-cancer is still questionable and needs more exploration in the future.

Another interesting result is the TIGIT-related functions and pathways. Our work shows that high TIGIT was associated with an enhanced function of apoptosis. This is consistent with the previous study. Kong et al. focused on TIGIT expression in T cells in patients with acute myelogenous leukemia (AML) (6). They confirmed the correct correlations between apoptosis and exhaustion of CD8^+^ T cells and the TIGIT, and the enhanced apoptosis or exhaustion could be reversed after the knockdown of TIGIT (6). Also, Song et al. demonstrated the significant role of TIGIT in aging CD8^+^ cells in aged mice (40), found that TIGIT was associated with high levels of expression of other inhibitory receptors, including PD-1 and showed features of exhaustion such as downregulation of the key costimulatory receptor CD28, the representative internal transcriptional regulation, the low production of cytokines, and high susceptibility to apoptosis. Importantly, their functional defects associated with aging could be reversed by TIGIT knockdown (40). Thus, TIGIT has great potential as a therapeutic target that several significant functions, such as apoptosis, could be reversed after targeting TIGIT.

Studies of TIGIT in NK cells can better show the important role of TIGIT in inhibiting anti-tumor immunity. Previous studies have shown that PVR molecules expressed on the surface of tumor cells can bind to TIGIT on the surface of NK cells, which lead to inhibitory signals in NK cells, and then reduce the function of NK cells to kill tumor cells (8, 41, 42). These results indicate that TIGIT is also an inhibitory molecule on the surface of NK cells. Moreover, exhaustion NK cells highly express TIGIT rather than PD1, and whether it is to knock out the TIGIT gene or to inhibit TIGIT with anti TIGIT antibody can increase the expression of CD107a, tumor necrosis factor (TNF), and other tumor suppressor factors in NK cells, enhance the tumor-killing ability of NK cells and prolong the survival time of tumor-bearing mice (8). Also, it is surprising that the specific knockout of the TIGIT gene in NK cells can reverse the depletion of NK cells and significantly reduce the expression of PD1 in tumor-infiltrating cytotoxic T cells (8). Manieri et al. systematically summarized the important mechanisms of TIGIT in inhibiting anti-tumor immunity (43), which mainly includes the following three mechanisms: first, the PVR of tumor cells or dendritic cells binds to the TIGIT on the surface of tumor-infiltrating CD8^+^T cells or NK cells, directly inhibiting the activity of these two immune cells. Second, TIGIT can also be used as a ligand. TIGIT ligands on the surface of tumor-infiltrating CD8^+^T cells or Tregs can bind to PVR receptors of tumor cells or dendritic cells, promote the production of anti-inflammatory cytokines such as IL-10 and inhibit the immune response. Third, the TIGIT on the surface of tumor-infiltrating CD8^+^T cells competitively binds to the PVR on the surface of tumor cells or dendritic cells, resulting in the failure of T cell-activated receptor CD226 to bind to PVR, thus inhibiting the activity of T cells (43).

These results indicate that targeting TIGIT can play a role in multiple ways and relieve the immunosuppression. This also inspires the combination therapy of PD1/PD-L1 and TIGIT monoclonal antibody. Johnston et al. reported that the combined use of TIGIT antibody and PD-L1 antibody at the same time is far better than blocking TIGIT or PD1/PD-L1 pathway alone, which can more significantly reduce the tumor volume and the survival time of tumor-bearing mice (5). Besides, CITYSCAPE (44), a randomized, double-blind, placebo-controlled phase II clinical trial of anti-TIGIT antibody tiragolumab combined with atezolizumab in the first-line treatment of patients with PD-L1 positive non-small cell lung cancer, demonstrated that the objective response rate (ORR) of combination therapy was 31.3%. In comparison, that of PD-L1 antibody monotherapy combined with placebo was 16.2%. Besides, in patients with high expression of PD-L1, ORR of combination therapy was 55.2%, while ORR of PD-L1 antibody monotherapy combined with placebo group was 17.2% (44). This is quite encouraging. As a result, Roche TIGIT monoclonal antibody tiragolumab has been recognized by FDA as a breakthrough therapy designation and combined with PD-L1 monoclonal antibody atezolizumab for the first-line treatment of metastatic non-small cell lung cancer with high expression of PD-L1 and non-EGFR nor ALK mutation patients.

TIGIT antibody showed huge potential in futural immunotherapy, and our works also identified TIGIT as an essential prognosis related and immune suppressive factor in KIRC. We discovered a significant correlation between PD1, PD-L1, and CTLA4 expression and TIGIT expression, which might give the explanation on the low response for the common immune monotherapy and might contribute to the combined therapy of PD1/PD-L1 or CTLA4 antibody therapy with TIGIT antibody in KIRC in the future. Besides, we found the expression of TIGIT was positive associated with the drug sensitivity of sunitinib, which might contribute to the combined therapy of the TIGIT antibody with sunitinib in KIRC in the future. More importantly, we discovered two potential drugs targeting TIGIT: Selumetinib and PD0325901. Interestingly, Selumetinib, a selective MEK1 inhibitor, was reported to enhance the antitumor activity of everolimusa against renal cell carcinoma by decreasing p-RPS6 and p-4E-BP1 dramatically, which caused G1 cell cycle arrest and preventing reactivation of AKT and ERK (45). Besides, Zeng et al. reported everolimus-induced autophagy involves activation of the ERK, which could impair the cytotoxicity of everolimus in RCC cells and inhibit the activation of ERK pathway-mediated autophagy like combined use of Selumetinib, which contributed to overcoming chemoresistance to everolimus (46). As for PD0325901, Diaz-Montero has claimed the combined use of PD0325901 contributes to abrogating the sunitinib resistance and leading to improved anti-tumour efficacy renal cell carcinoma (47). Thus, based on these studies and our discoveries, combined therapy of TKIs with Selumetinib or PD0325901 also shows great potential in treating KIRC in the future. More in-depth cohort studies were urgently needed in the future.

There are several limitations in this study. Firstly, our analysis were based on the bulk RNA-seq. However, the results would be more precise if the data were acquired by single-cell sequencing, which could contribute to our understanding of TIGIT in different cell types. Secondly, we suggested several novel therapeutic strategies for KIRC in this research, such as the application of Selumetinib or PD0325901 monotherapy as targeting TIGIT, combined therapy of PD1/PD-L1 antibody with TIGIT antibody, combined therapy of sunitinib with Selumetinib or PD0325901, et al. They were all hypotheses, and we need carrying out further studies including laboratory experiments and real-world cohort studies in the future.

## Conclusion

TIGIT plays an essential role in tumorigenesis, progression in KIRC. High expression of TIGIT results in poor survival of KIRC and higher drug sensitivity to sunitinib and anti-PD1 immunotherapy. Besides, Selumetinib and PD0325901 may be potential drugs targeting TIGIT, and combined therapy of anti-TIGIT and other treatments show great potential in treating KIRC.

## Data availability statement

The original contributions presented in the study are included in the article/**Supplementary Material**. Further inquiries can be directed to the corresponding authors.

## Author contributions

Conception and design: Q-DX, BL, WG, HL, and S-GW. Acquisition of data: Q-DX and BL. Analysis of data: Q-DX and BL. Interpretation of data: Q-DX, BL, Drafting the manuscript: Q-DX and BL. Revising the manuscript: All authors. All authors contributed to the article and approved the submitted version.
