# Supplementary material for: Integrated bioinformatic analysis and cell line experiments reveal the significant role of the novel immune checkpoint TIGIT in kidney renal clear cell carcinoma
Source: Front Oncol. 2023 Mar 24;13:1096341. doi: 10.3389/fonc.2023.1096341 (PMC10079921; doi:10.3389/fonc.2023.1096341)
Supplement: Supplementary file 1 [file Table_1.docx]

**Supplementary Table S1: qRT-PCR primers used in the study**

| Name | Sequence |
| --- | --- |
| *GAPDH* | Forward: 5′-CCATGTTCGTCATGGGTGTG-3′ |
|  | Reverse: 5′-CAGGGGTGCTAAGCAGTTGG-3′ |
| *TIGIT* | Forward: 5′-TGGTCGCGTTGACTAGAAAGA-3′ |
|  | Reverse: 5′-GGGCTCCATTCCTCCTGTC-3′ |
